# Mycotoxin Contamination Status of Cereals in China and Potential Microbial Decontamination Methods

**DOI:** 10.3390/metabo13040551

**Published:** 2023-04-12

**Authors:** Jing Zhang, Xi Tang, Yifan Cai, Wen-Wen Zhou

**Affiliations:** 1 College of Biosystems Engineering and Food Science, Ningbo Research Institute, Zhejiang University, Hangzhou 310058, China; 2 School of Chemical and Biomolecular Engineering, The University of Sydney, Sydney, NSW 2006, Australia

**Keywords:** cereal contamination, mycotoxins, China, microbial decontamination mechanism

## Abstract

The presence of mycotoxins in cereals can pose a significant health risk to animals and humans. China is one of the countries that is facing cereal contamination by mycotoxins. Treating mycotoxin-contaminated cereals with established physical and chemical methods can lead to negative effects, such as the loss of nutrients, chemical residues, and high energy consumption. Therefore, microbial detoxification techniques are being considered for reducing and treating mycotoxins in cereals. This paper reviews the contamination of aflatoxins, zearalenone, deoxynivalenol, fumonisins, and ochratoxin A in major cereals (rice, wheat, and maize). Our discussion is based on 8700 samples from 30 provincial areas in China between 2005 and 2021. Previous research suggests that the temperature and humidity in the highly contaminated Chinese cereal-growing regions match the growth conditions of potential antagonists. Therefore, this review takes biological detoxification as the starting point and summarizes the methods of microbial detoxification, microbial active substance detoxification, and other microbial inhibition methods for treating contaminated cereals. Furthermore, their respective mechanisms are systematically analyzed, and a series of strategies for combining the above methods with the treatment of contaminated cereals in China are proposed. It is hoped that this review will provide a reference for subsequent solutions to cereal contamination problems and for the development of safer and more efficient methods of biological detoxification.

## 1. Introduction

Cereal farming has always been a major part of human agricultural production, as cereals are essential human foods and animal feed resources. Based on global survey data and thresholds, Marin et al. [1] confirmed the Food and Agriculture Organization’s (FAO) claim that about 25% of global cereals are contaminated with mycotoxins. Mycotoxins are secondary metabolites produced by certain fungi in oilseeds, cereals, legumes, nuts, and processed products during the pre-harvest and post-harvest stages [2]. The common mycotoxins include aflatoxins (AFs), ochratoxin A (OTA), zearalenone (ZEN), deoxynivalenol (DON), T-2 toxin (T-2), and fumonisin (FB), all of which exhibit a high melting point, poor solubility, a long half-life, and are carcinogenic, mutagenic, and teratogenic [3]. The International Agency for Research on Cancer (IARC) has classified many natural mycotoxins as being linked to carcinogenicity [4] and recognized aflatoxin as the most serious carcinogen as it has the strongest biological toxicity. Specifically, AF exposure in combination with hepatitis B virus infection increases the risk of liver carcinogens (hepatocellular carcinoma) in some areas of South Africa and China [5,6]. Histological analysis has confirmed that cattle mortality due to liver damage is strongly associated with the consumption of AF-contaminated peanuts [7]. In addition, continuous exposure to high doses of aflatoxin can cause growth retardation in children [8]. Additionally, ochratoxin, which is as harmful as aflatoxin, is often detected in pork for human consumption [9]. According to Petkova-Bocharova et al. [10], as a nephrotoxin, ochratoxin may be related to the Balkan endemic nephropathy and urinary tract tumors. Table 1 lists the mycotoxins commonly found in cereals and their effects.

The growing public attention paid to food quality and safety has strengthened the scientific research into mycotoxins and toxin-producing molds in cereals [30,31,32]. Developing countries generally face a greater threat than developed countries because of their inadequate storage and transportation facilities. Moreover, the U.S. Department of Agriculture reports that China’s total grain production reached 548.5 million tons in 2019–2020, accounting for about 20% of the world’s total production, among which more than 10 million tons of cereals were contaminated above national standards [33]. Therefore, the decontamination of mycotoxins in cereals remains a research hotspot.

The growth of molds and mycotoxin production cannot be separated from warm temperatures (28–31 °C) and high humidity (60–90%). The climate in China exhibits significant differences in rainfall, temperature, and humidity across its vast territory, accompanied by seasonality and an extreme climate, making cereals more susceptible to mold contamination [34,35]. Although established physical and chemical methods are widely used to decontaminate cereals, they often result in nutritional losses or chemical residues. Biological (e.g., microbial) decontamination methods present an alternative method for inhibiting the growth of foodborne pathogens and, importantly, keeping mycotoxin concentrations below the prescribed limits [36]. Some of these methods have the advantages of being efficient, economical, and environmentally friendly [37].

Recent developments in microbiology, such as the studies of Mannaa et al. and Simonetti et al. [38,39], which focus on important processes for the microbial decontamination of cereals outside China, have led to a renewed interest in microbial decontamination, indicating a growing trend toward replacing physical or chemical means with microbial methods. The reaction between the studied *Stenotrophomonas* species and eleven food and feed crops contaminated by trichothecene mycotoxins [40] triggered detoxification effects beyond the researchers’ expectations, highlighting the industrial potential of using such strains to reduce trichothecene contamination in food and feed and to minimize their cytotoxicity.

This review summarizes the status of mycotoxin contamination of major cereal crops, including rice, wheat, and maize, in 30 provincial areas of China between 2005 and 2021. Data were sourced from the Food and Agriculture Organization of the United Nations (FAO), the State Food and Material Reserve Administration, the State Administration of Market Supervision, and the World Health Organization (WHO) databases. The review also analyzes the persistent problems with the established physical and chemical detoxification methods. Against this background, our study proposes practical methods for inhibiting or detoxifying of mycotoxins using microorganisms or active microbial substances and systematically describes the mechanisms of different inhibition or detoxification methods. Finally, it discusses the opportunities and challenges in the practical application of various methods to solve the ongoing problem of mycotoxin contamination in China.

## 2. Cereal Contamination by Mycotoxins in China and the Existing Decontamination Methods

### 2.1. Impact of Climate Change on the Mycotoxin Contamination Rate of Cereals in China

China serves as a major global cultivator and preserver of numerous cereals, including maize, wheat, and rice [41]. Above 60% of consumers rely on cereals as their main energy source in this developing country [42]. Due to China’s distinct continental monsoon climate and complex geographical conditions, high temperature, high humidity, and drought are observed in many of its regions annually [43]. These conditions benefit the growth and metabolism of toxin-producing molds [44,45].

**Figure 1:**
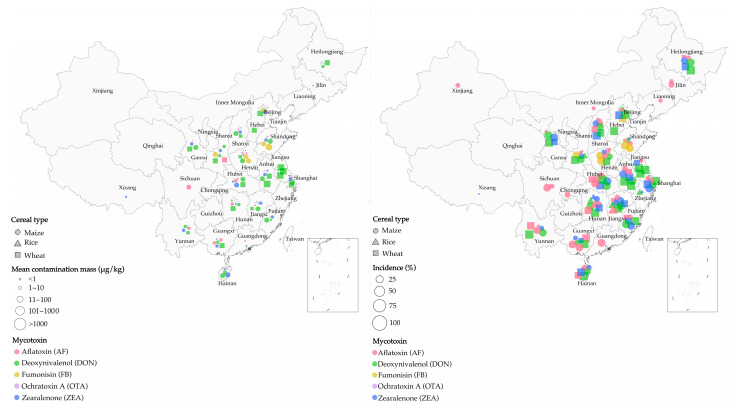
Mycotoxin contamination of cereals (maize, rice, and wheat) in surveys across China. (**Left**) mean mass of cereal contamination in different regions; (**right**) incidence of cereal contamination in different regions. (Source: our own study.)

The amount of cereal contaminated by mycotoxins increased due to climate change in southeastern China from 2009 to 2015 [46,47,48]. DON is a mycotoxin secreted by *Fusarium* species, including *F. culmorum, F. pseudograminearum*, and *F. graminearum*, causing *Fusarium* head blight (FHB) in cereals [49,50]. Their production has been reported to be associated with increased rainfall and higher temperatures [45,46]. From 2010 to 2012, increased rainfall led to severe DON contamination of wheat in Jiangsu Province (Southeastern China) [51]. Additionally, an increasing trend in DON-contaminated maize (*n* = 50) was observed in Shanghai for 4 consecutive years (from 2009 to 2012), with an average contamination concentration of 130 μg/kg [52]. The above observations demonstrate the strong correlation between AF-contaminated cereals and temperature and humidity in China. Specifically, most molds can multiply at a relative humidity above 70% [53], with *Aspergillus flavus* being able to multiply consistently at water contents ranging from 94 g/kg to 175 g/kg and temperatures in the range of 30–40 °C [54]. A 2010 survey revealed that the better growth conditions for *A. flavus* and *A. parasiticus* Speare in Huaian and Fusui (Southeastern China), as compared to Huantai (Northeastern China), were caused by the lower annual rainfall and temperature in the former regions. This in turn explained the greater amount of aflatoxin B_1_ (AFB_1_)-contaminated maize in Huaian and Fusui [47], with >20 μg/kg AFB_1_ in ~35% of maize samples collected in Huaian [55]. Figure 1 illustrates our review of cereal contamination by mycotoxins in China as calculated by several surveys (a total of 8700 samples). Combined with other findings [56,57,58,59,60,61], this overview demonstrates that the humid and hot climate in southern China is favorable for mycotoxin production by fungi, resulting in more severe cereal contamination in southern China than in the north [41].

### 2.2. Correlation between Cereal Types and Mycotoxin Contaminants in China

It is common for specific mycotoxins to only be found in certain cereals in China. For example, among 151 rice samples tested in 3 northeastern provinces of China, the contamination rate of AF reached 63%, while the contamination rate of OTA was only 5.3% [62]. Fumonisin B_1_ (FB_1_), secreted by *F. moniliforme*, was mainly contaminated in maize and its products when the ambient humidity was 18–23%. Figure 1 shows the contamination of maize with FB_1_ in Henan and Shandong provinces. In warm and humid areas, *Alternaria* species produce tenuazonic acid (TeA), which is the main mycotoxin present in wheat samples [63,64]. Overall, DON and OTA were found in oat, barley, and gypsophila. AF and ZEN were frequently found in maize, while OTA, T-2, HT-2, and diacetoxyscirpenol (DAS) were not specific to contaminated cereals [60,65,66]. Figure 1 shows the cereals contaminated with different mycotoxins found in various regions of China.

### 2.3. Existing Mycotoxin Detoxification Methods

In China, mycotoxins in food and feed are mainly removed using established physical and chemical methods. Specifically, physical methods combine light, ultrasound, or ultra-high temperatures with the sorting, washing, and milling process [67], or use UV/gamma radiation [68] during cereal processing to remove mycotoxins, but this may lead to nutrient loss in the food. Chemical methods involve the addition of substances that promote mycotoxin degradation during cereal processing, such as oxidants, ammonium, sodium hydroxide, and diatomite. However, these substances are hard to remove and can remain in cereals [69,70,71]. Moreover, the application of organic solvents for extraction generates wastes and is not environmentally friendly. The advantages and disadvantages of these two approaches for detoxification are analyzed in Table 2. Considering the chemical and thermal stability of most mycotoxins, complete detoxification (i.e., mitigation of their toxicity by altering or shifting the relevant structure) cannot be achieved using conventional measures. What is more, mycotoxin contamination is usually heterogeneous, which poses another challenge for the established treatment methods.

During the storage of cereal, toxin-producing molds reproduce rapidly, allowing mycotoxins to accumulate in cereals; this causes significant potential economic losses. For example, one study tested 182 cereal samples from cereal silos in Hubei Province, China, and showed that the average content of FB_1_ was 12.55 mg/kg [79]. To avoid the huge losses caused by mycotoxins in cereals, the Chinese government has updated the thresholds for common mycotoxins in cereals and other agricultural commodities. Manufacturers in food and feed processing should follow the relevant standards in Table 3. In 2019–2020, the Ministry of Agriculture of China issued the National Agricultural Product Quality and Safety Risk Assessment Plan, which took the contamination of common mycotoxins in cereals in different regions of China as a key assessment item. These decisions indicate that mycotoxin contamination in the production and storage of cereals in China had not been effectively controlled previously. Furthermore, in consideration of the serious hazards caused by mycotoxins due to their high toxicity, scientists have recommended enhanced mold protection from early planting to storage and the adoption of more effective detoxification methods [43,80].

## 3. Microbial Methods of Inhibiting Mycotoxin Growth and Detoxifying Mycotoxins

### 3.1. Decontamination Methods Based on Microorganisms

The most recent research focus in the field of cereal contamination is the use of microbial methods to inhibit the growth of toxin-producing molds and detoxify mycotoxins. The microorganisms employed include yeasts, lactic acid bacteria, bacilli, non-toxic molds, and marine microorganisms. The microorganisms that play a crucial role in the prevention and control of toxic mold and mycotoxin production in cereals are discussed here, and they are classified according to their different mechanisms.

#### 3.1.1. Adsorption and Binding Using Microorganisms

The physisorption of some microorganisms, especially *Saccharomyces cerevisiae* strains, can reduce certain mycotoxins in feed and cereals. Stanley et al. [81] demonstrated improved poultry growth after the addition of yeast to AF-contaminated feed. Another study [82] pointed out that the active component of yeast for mycotoxin adsorption is the glucomannan in the yeast cell wall, so both yeast [83] and the isolated yeast cell wall can act as mycotoxin adsorbents. In addition, the modification of yeasts can increase the noncovalent interaction between the side chains of cell walls and toxin molecules. One study demonstrated that yeast modified with β-1,3-glucan adsorbed 81.6%, 27.8%, and 25.6% of AFB_1_, T-2, and OTA, respectively [84]. Yiannikouris et al. [85] extracted yeast cell walls and tested their adsorption capacity using *S. cerevisiae* as the raw material. They observed that the affinity of β-D-glucan for different toxin molecules was in the order of AFB_1_, DON, and OTA from high to low. Similarly, some yeasts modified by the crosslinking-esterification of alkyl ammonium ion glucan compounds had a high adsorption capacity for ZEN (183 mg/g) and T-2 (10 mg/g) [86]. Accordingly, various mycotoxin adsorbents have been developed using yeast cells to reduce the harmful effects of mycotoxins.

Scientists have conducted many studies on the binding sites of lactic acid bacteria and probiotics to mycotoxins, as lactic acid bacteria and several probiotics can be powdered and added to mycotoxin-contaminated cereals to remove mycotoxins [87]. An in vitro comparison of AFB_1_ binding to *Lactobacillus* and *Propionibacterium* revealed that *Lacticaseibacillus rhamnosus* GG and *L. rhamnosus* LC705 could bind nearly 80% of AFB_1_ (5 μg/mL) within one hour at 10^10^ cfu/mL [88]. After heat inactivation and acid treatment, the two *Lactobacillus* strains are more effective in reducing AFB_1_ and exhibit a better binding capacity for ZEN and its derivative α-ZOL [89]. Accordingly, heating and acid treatment may affect the binding sites of probiotics, thereby increasing their binding affinities for AFB_1_ and reducing its accumulation. Niderkorn et al. [90] found that *L. rhamnosus* GG, *L. delbruekii* ssp. *bulgaricus* R0149, and *Leuconostoc mesenteroides* R1107 could detoxify ZEN, FB, and several trichothecenes (DON, nivalenol, and T-2), revealing that peptidoglycans in the bacterial cell wall are binding sites for Aspergillus toxins. Other researchers demonstrated that the peptidoglycans of *L. rhamnosus* GG [91] and the teichoic acid of the *Lacticaseibacillus casei* strain Shirota [92] are indispensable components for their cell walls binding to AFB_1_. They are involved in the formation of a reversible complex between the mycotoxin and the microbial surface, and participate in the process of mycotoxin binding and release [93].

Although the addition of appropriate probiotics to cereals can reduce the number of mycotoxins [94], the application of this approach in food and other commodities is limited. This is because many lactic acid bacteria that are generally regarded as safe (GRAS) as bioconjugates require strict anaerobic characteristics and abnormal culture conditions. It is clear that the future development of multiple safe strains for incorporation into contaminated cereals remains challenging. Additionally, more microbial adsorption and binding mechanisms need to be investigated to enable the further exploration of more efficient microbial detoxification pathways.

#### 3.1.2. Biocompetitive Inhibition Using Microorganisms

Biocompetitive inhibition is based on the inoculation of highly competitive non-toxic strains in the soil where the cereal grows, competing with the toxin-producing molds. The non-toxic strains, which are generally from the same species as the toxic strains, are able to reduce or inhibit mycotoxin production to some extent, thus reducing the probability of cereal infection. Such biocompetitive methods were first used by the ARS laboratories in the USA to reduce AFs in cereals [95]. Since then, numerous studies have also demonstrated that non-toxic *Aspergillus* spp. can be used to reduce Afs in cereals and crops by competing biologically with AF-producing species and inhibiting the latter’s metabolism. Dorner et al. [80] used competition among microorganisms to inoculate the non-toxic *A. parasiticus* into peanut-producing land to reduce AFB_1_ content in edible peanuts by 83–98%. Considering the biocompetitive relationships among microbes, another study [96] introduced the *Aspergillus* strain (atoxigenic) into the soil, which decreased AFB_1_ content in cotton seeds compared to the control group. The inoculation of corn with non-toxigenic *A. flavus* K49 and CT3 reduced AFs by 65–94% [97]. Additionally, non-toxic *A. flavus* NRRL 18543 and NRRL 21882 are also commercially produced nowadays, due to their highly effective removal of mycotoxins from cereals including corn [97,98,99]. Moreover, antagonistic bacteria can inhibit the growth and infection of toxic fungi. For example, *Bacillus subtilis* and some species of the genus *Streptococcus* were employed to control wheat scab, and *Bacillus polymyxa* AFR0406 was shown to prevent wheat scab and sheath blight [100]. Such strains are highly effective in inhibiting mycotoxin production. Although they are able to inhibit the growth of toxin-producing molds under laboratory conditions, their ability to achieve good results in cereals and foods is limited due to the difficulty of bringing bacterial cells to the site of infection. On the other hand, although the purpose of using the biocompetitive method is to find more favorable strains than toxigenic molds, the competition mechanism is still unclear, making it impossible to determine the amount of inoculum required and the suitable treatment conditions.

### 3.2. Microbial Active Substance Decontamination Methods

Microorganisms can purify cereals by generating active substances or degrading mycotoxins into less toxic or non-toxic substances. The following sections describe these two processes, providing theoretical support for this approach to reducing mycotoxins in cereals.

#### 3.2.1. Inhibition Using Microbial Active Substances

Microorganisms inhibit the growth of toxin-producing molds, and mycotoxins exert toxicity by producing active substances such as secondary metabolites. Table S1 lists many active substances and their action conditions, including polypeptides, small molecular substances, enzymes, and organic acids [101]. Some metabolites of yeast and lactic acid bacteria (e.g., 2-phenylethanol, phenyllactic acid, and indole lactic acid) have antagonistic effects [102,103,104,105,106,107,108]. Multiple antifungal compounds contained in the supernatant of *Lactiplantibacillus plantarum* K35 completely inhibit the growth and AF production of *A. flavus* TISTR3041 and *A. parasiticus* TISTR3276 [108]. Munimbazi et al. [109] found that the fermentation broth of *B. pumilus* inhibited the production of AF. Later, the same authors identified cyclic polypeptides or nonpeptide compounds in the broth as the active ingredient isolated from *B. pumilus* [110]. When the concentration of the active substance was 0.2 mg/mL, its inhibition rate of OTA and *A. ochraceus* NRRL 3174 hyphae was approximately 71% and 76%, respectively. Futhermore, aflastatin, an active substance with antibiotic effects extracted from *Streptomyces*, was able to prevent the production of Afs by *A. parasiticus* at a concentration of 0.5 μg/mL [111]. In addition to the active substances extracted from microbial cultures, volatile compounds produced during the metabolism of certain microorganisms can also purify cereals. For example, phenyl ethanol and 1-pentanol, produced by *Enterobacter asburiae* Vt-7, have been shown to downregulate the expression of AF genes and thus significantly reduce *A. flavu* contamination of cereals [112]. Studies have also demonstrated that the main volatile compound produced by the four yeasts (*Cyberlindnera jadinii* 273, *Candida friedrichii* 778, *Candida intermedia* 235, and *Lachancea thermotolerans* 751) is 2-phenylethanol, which makes a significant contribution to the inhibition of growth and OTA production by *A. ochraceus* and *A. carbonarius* [102]. Interestingly, the mechanism of inhibition by 2-phenylethanol is similar to that of some *Streptomycete* spp. in that it inhibits the spore production of toxin-producing molds [107]. Although various microorganisms produce active substances that can inhibit the growth of toxin-producing molds and halt mycotoxin production, the conditions of action for these active substances are complex and varied, posing a significant challenge to the practical application of such substances.

#### 3.2.2. Detoxification Using Microbial Active Substances

In addition to inhibition, detoxification with microbial active substances is one of the main strategies for reducing the contamination of cereals with mycotoxins [113,114,115,116,117,118,119,120,121,122]. Table 4 lists the detoxification conditions and products and detoxification efficiency of various microorganisms. According to the study conducted by Takahashi-Ando et al. [123], the lactone hydrolase ZHD101 produced by *Clonostachys rosea* IFO 7063 can bind and break the lactone ring of zearalenone. Meanwhile, monooxygenases can oxidize the 12,13-epoxy group of deoxynivalenol. The functional group of mycotoxins are cleaved to form new metabolites that are more easily excreted by the digestive system, which significantly reduces their toxic effects. The active substances produced by specific microorganisms, such as epoxidase, extracellular xylanase, proteases, and esterases, can detoxify DON and ZEN [124,125]. Other active components in cell-free cultures have also been confirmed to detoxify mycotoxins [126,127,128]. According to Teniola et al. [103], cell-free extracts of four species of bacteria (*Rhodococcus erythropolis* DSM 14303, *Nocardia corynebacterioides* DSM 12676, *Nocardia corynebacterioides* DSM 20151, and *Mycobacterium fluoranthenivorans* sp. nov. DSM 14304) substantially detoxified AFB_1_ at 30 °C, pH = 7.0. There are relatively few studies on the use of active microbial substances for detoxification, leading to limitations in the further development of efficient detoxification procedures using this approach.

### 3.3. Other Microbial Inhibition Methods

A great leap in molecular biology relates to the uncovering of the whole genome sequences of ten important *Aspergillus* species: *A. flavus*, *A. parasiticus*, *A. fumigatus*, *A. sojae*, *A. nomius*, *A. tamarii*, *A. pseudotamarii*, *A. bombycis*, *A. oryzae*, and *A. sojae* [153], which allowed scientists to explore the biochemical mechanisms of mycotoxin synthesis. A comparison of the genomes of toxin-producing molds with those of similar strains without toxin-producing activity will help to identify the key toxin-producing genes and corresponding enzymes and proteins. Then, the key genes involved in mycotoxin synthesis can be removed by gene knockout, and the resulting non-toxic mold can replace the original toxin-producing strain and alleviate mycotoxin contamination in cereals [154]. Cereals can be genetically modified to enhance the expression of endogenous genes that resist toxin-producing molds and mycotoxin contamination. Additionally, transgenic technology can be used to introduce exogenous genes, thus equipping cereals with antimicrobial properties and reducing mycotoxin contamination [155].

The practical production of the enzyme is difficult due to the complex separation and purification process, its unstable activity, and the harsh survival conditions. This limitation can be overcome by cloning detoxification genes with high activity and by achieving the heterologous expressions of degrading enzyme genes in prokaryotic or eukaryotic engineered strains [156,157] (more information on mechanism (5) is provided in Section 3.4).

Combination enzymes with microorganisms antagonistic to the toxic strain can also increase enzymatic activity and improve the decontamination of mycotoxins [158]. Zuo’s research demonstrates that the addition to chicken feed of probiotics and the AFB_l_ degrading enzyme conjugates resulted in a reduction in toxic substances and a significant improvement in the antioxidant capacity of chicken liver cells and the chicken production performance [159]. However, only a few degradation enzymes have been found to be efficient in degrading mycotoxins. In the future, a larger number of strains need to be screened and studied in depth for their degradation ability, enabling scientists to isolate and purify their degradation enzymes and find the genes that regulate these enzymes. Then, these enzymes will be cloned into heterologous expression vectors so that they can be efficiently used in the actual production of cereals and feed. Furthermore, since bioactive enzymes are highly specific for mycotoxins, efforts should be made to explore appropriate degrading enzymes for rare or even unknown mycotoxins [160,161]. 

These studies reveal that molecular biotechnology can identify the genes and enzymes responsible for mycotoxin synthesis and transfer genes encoding good mycotoxin degradation properties. Further understanding of the mechanisms of and interactions between toxins and microorganisms will provide a significant theoretical and practical basis for controlling mycotoxins in cereals using biotechnology.

### 3.4. Mechanism of Mycotoxin Reduction Using Microbiological Methods

**Figure 2:**
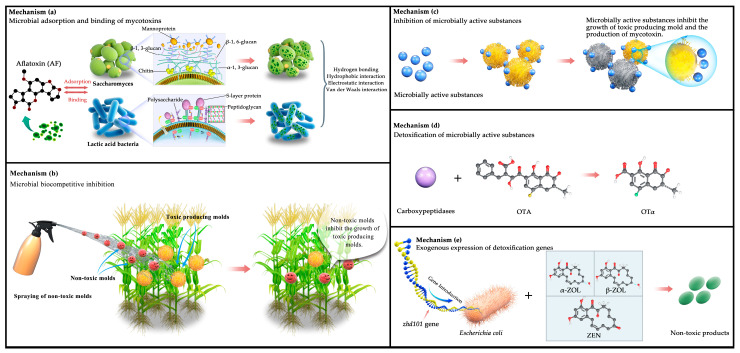
Mechanisms of mycotoxin reduction. (**a**) Microbial adsorption and binding of mycotoxins; (**b**) microbial biocompetitive inhibition; (**c**) inhibition of microbially active substances; (**d**) detoxification of microbially active substances; (**e**) exogenous expression of detoxification genes. (Source: our own study).

Figure 2 illustrates five mechanisms of mycotoxin reduction: (a) previous studies have demonstrated a direct relationship between the cell walls of beneficial microorganisms (e.g., yeast and lactic acid bacteria) and their adsorption and binding of mycotoxins [162,163]. Specifically, the yeast cell wall consists of three layers: the outer layer with mannose oligosaccharides and protein complexes, the middle layer with dextran, and the inner layer with chitin. Luo et al. [164] reported that the structural framework formed by β-1,3-glucan and chitin in the yeast cell wall provides more meshes for the adsorption of mycotoxins. The higher the density of the meshes, the stronger the adsorption capacity. This structure provides various hydrogen bonding, electrostatic, and hydrophobic interaction sites for mycotoxin adsorption, which enhances the adsorption capacity, as well as contributing to the purification of cereals and feeds. Similarly, peptidoglycans, polysaccharides, and S-layer proteins in the lactic acid bacteria cell wall contribute to the mycotoxin-binding process [91,165], and they generate chemical complexes with mycotoxins via hydrogen bonds, van der Waals forces, and hydrophobic interactions [166,167]. (b) Biocompetition using non-toxic molds is shown in the figure. The atoxigenic strains compete against the toxin-producing molds for nutrition, limiting the latter’s access to appropriate spaces and exhausting nutrients in the wound site. This causes the toxin-producing molds to stop growing due to a lack of nutrients. (c) Microbial active substances can specifically bind to toxin-producing molds, induce oxidative stress, break biochemical reactions, and block the physiological metabolism of certain sensitive fungi, thereby inhibiting the conidial growth and mycotoxin production of these molds. (d) Detoxifying mycotoxins using active microbial substances involves specific, affinity, and high-catalytic enzymes that convert mycotoxins into small non-toxic or less toxic molecules. It involves acetylation, ring cleavage, hydrolysis, glycosylation, deamination, and decarboxylation [143,144,151,152]. For example, *Yarrowia lipolytica* Y-2, *Acinetobacter* sp. *neg1* ITEM 17016, and *B. amyloliquefaciens* ASAG1 produce carboxypeptidase, detoxifying ochratoxin under appropriate conditions and producing the less-toxic OTα [146,147,148]. (e) The removal of key genes for mycotoxin synthesis or the enhanced expression of degradation enzyme genes is another method for decreasing mycotoxin levels in cereals. *Zhd101* is a new gene encoding a hydrolase of inhibitory metabolites for ZEN [168]; it can be introduced into *Escherichia coli* and *S. cerevisiae* to break down ZEN, α-zearalol, and β-zearalol into non-toxic products within 24 h.

## 4. Conclusions and Prospects

In recent years, an increasing amount of research has explored the methods for the mycotoxin decontamination of Chinese cereals and agricultural products [169,170], indicating that the problem still needs to be solved. This review discussed the status of cereal contamination in China. The collected data from 2005 to 2021 show that mycotoxin levels in many cereals still exceed national standards. This is due to the temperature and humidity in China being favorable for the growth of toxin-producing molds, especially in southern China, which has a variable climate and complex geography. Physical and chemical decontamination methods have certain limitations, such as insufficient cereal nutrition, poor safety, and high energy consumption. To further reduce the content of mycotoxins in cereals and avoid the shortcomings of the established methods, it is imperative to develop new methods. This paper presents a variety of practical approaches to biotechnological detoxification, including the microbial adsorption and binding of mycotoxins, the competitive inhibition of the growth of toxin-producing molds or mycotoxin production, or the specific screening of certain microorganisms or enzymes to detoxify or produce non-toxic degradation products by destroying or modifying mycotoxins with the participation of their secondary metabolites or secreted intracellular and extracellular enzymes [171,172]. Furthermore, some previous studies were used to systematically summarize the mechanisms of various biotechnological detoxification methods, providing a more intuitive reference for preventing and controlling cereal contamination in China. Combining these findings with the specific conditions of the Chinese environment is expected to reduce mycotoxin contamination in grains during processing, transportation, and storage. This will reduce the health risks to consumers and the economic losses to the feed industry and animal husbandry.

Based on the expectations outlined in the previous section, the following recommendations are made to ensure that the detoxification process becomes more effective and environmentally friendly: (1) the summer season in southern China is characterized by a temperature of ~30 °C and relatively high humidity, creating the right conditions for active substances. Therefore, depending on the temperature and humidity of the environment, different kinds of microorganisms can be cultivated, and many active substances are produced, which in turn detoxify or inhibit the production of mycotoxins, thus reducing the contamination of cereals. (2) Northern regions of China such as the Liaoning and Jilin Provinces have a dry climate and low temperatures. In these regions, more *Spirulina* sp. and *B. pumilus* can be used for cereal detoxification, considering the inhibition conditions of microorganisms presented in Table S1 and Table 4; they will have a good detoxification effect on the main mycotoxins. (3) The typical climatic characteristics of the area north and east of the Qinling–Huaihe line limit the main cereals grown to wheat and corn, which are susceptible to AF and DON infections, respectively. Therefore, spraying active microbial substances produced by *Bacillus* sp. and *Spirulina* sp. on cereals will likely yield more effective detoxification effects. (4) Given that the southern region is contaminated with multiple mycotoxins due to the diversity of cereal varieties and frequent rainy seasons, it may be impractical to utilize active substances that specifically degrade one mycotoxin. This prompts the use of multiple probiotic microorganisms to achieve detoxification by the biocompetitive inhibition of toxin-producing molds or adsorption of mycotoxins by beneficial bacteria, such as by using yeast in agricultural fields or barns.

The development of mycotoxin biodegradation and the inhibition of toxin-producing molds has positive effects on the detoxification of cereals and other agricultural commodities. However, several challenges and limitations remain: (1) the current research on microorganisms for adsorption and combined detoxification mainly focuses on yeast and its cell wall, lactic acid bacteria, and other strains, which still cannot meet the needs of cereal food safety. (2) The mode of action of antagonistic microorganisms is unknown, limiting the use of these organisms in field conditions. (3) The complex and diverse conditions of the inhibition of active substances produced by various microorganisms and the relatively few studies that use them for detoxification pose challenges to the effectiveness of cereal detoxification and the further development of efficient detoxification products. (4) There are few studies on degradation enzymes that effectively degrade mycotoxins, posing a challenge to the isolation and purification of degradation enzymes released by microorganisms. In conclusion, much work needs be done to address the current limitations and challenges so that microbial detoxification techniques can be fully used to detoxify cereals and agricultural products. First, since strains have different detoxification effects on different mycotoxins, using a single strain often does not achieve the desired effect. Therefore, it is possible to further improve the detoxification effect by combining multiple strain detoxification methods in future research. Second, most strains currently screened in the natural environment have detoxification effects on cereals but still do not meet the GRAS Act standards. Scientific safety studies could be conducted on the strains known to have detoxification effects so that most strains can be legally added to cereals as soon as possible. Finally, a large number of strains with detoxification abilities can be further screened, the mechanism can be studied in depth, the degradation enzymes released by the strains can be further isolated and purified, the genes regulating the degradation enzymes can be searched for, and then further cloning can be performed to finally achieve efficient expression in vector. In the future, we hope to combine scientific research with Chinese environmental conditions and cereal storage methods to solve the problem of the mycotoxin contamination of cereals and crops as early as possible.

## Figures and Tables

**Table 1:** Common mycotoxins in cereals and their toxicology.

Mycotoxin (Toxic Dose)	Food Commodity	Toxicology	
		Human	Animals
Aflatoxins (AFs) (>2 ppm or 300 μg/kg)	Maize, sorghum, pearl millet, rice, wheat, oilseeds, spices, and tree nuts	1. Ingestion of medium or high doses of AFs can cause acute liver damage, potentially leading to death. Chronic AF poisoning can lead to cirrhosis and liver cancer [11].	1. Increased mortality, reduced hatchability, growth rate, meat and egg yield, and quality in poultry [12].
		2. Neurotransmitter deficiency impairing the central nervous system [13].	2. In dairy cows and beef cattle, acute AF poisoning can decrease milk production and cause weight loss and death [14].
		3. Immunosuppression [15].	
Ochratoxin A (OTA) (>100 ng/g)	Rice, barley, maize, wheat, flour, and bran	1. Balkan endemic nephropathy (BEN) and chronic interstitial nephropathy (CIN) [16].	1. Excessive intake of OTA can cause kidney disease in pigs. 2. In poultry, weight loss, decreased egg production and eggshell quality, and renal toxicity [17].
		2. Genotoxic and carcinogenic [18].	
Fumonisin (>200 μg/g)	Rice, wheat, peanut, barley, maize, rye, oat, and millet	1. Esophageal cancer, oral cancer, pharyngeal cancer, and fetal neural tube defects [19].	1. Pulmonary edema in pigs and leukoencephalomalacia in horses. 2. Toxic to the liver and kidneys of poultry and livestock [20].
		2. Promotes AF-initiated liver tumors [21].	
Zearalenone (ZEN) (>0.5 μg/kg)	Maize, rice, wheat, and barley	1. Increased risk of breast cancer [22].	Miscarriage and infertility in livestock [23].
		2. Excessive ZEN intake during pregnancy can reduce embryo survival [24].	
		3. Genetic, immune, blood, and liver toxicity [23].	
Citrinin (>20–40 mg/kg)	Wheat, rye, barley, and beans	1. Chronic poisoning can cause kidney failure and weight loss.	1. Slow growth and watery feces in poultry.
		2. Embryotoxic, immunotoxic, and teratogenic effects [25].	2. Chickens, rabbits, and mice experience varying degrees of liver and kidney damage [26].
Deoxynivalenol (DON) (>19.3 ng/g)	Wheat, barley, oats, rye, and maize	Excessive intake of DON can cause nausea, vomiting, diarrhea, abdominal pain, headache, and fever [27].	1. Excessive intake of DON can cause acute gastrointestinal diseases and immune dysfunction.
			2. Weight loss and anorexia [28].
T-2 toxin (>0.05–10 mg/kg)	Wheat, maize, barley, rice, soybeans, and oats	1. Skin blistering, pain, burning, itching, and inflammation.	1. Cows were diagnosed with gastroenteritis lesions, intestinal bleeding, decreased milk production, and no estrus cycle.
		2. Dyspnea and coughing after inhalation, as well as vomiting, diarrhea, and anorexia.	2. Poultry were diagnosed with impaired immune systems, damaged hematopoietic systems, and altered feather patterns.
		3. Genetically toxic and cytotoxic with adverse effects on the immune system [29].	3. Pigs were diagnosed with gastric bleeding, intestinal necrosis, and a refusal to feed [29].

**Table 2:** Physical and chemical detoxification methods with analysis.

Detoxification Approach	Specific Method	Pros	Cons	References
Physical	Sorting	Reduces aflatoxin contamination level by up to 70–80%.	Inefficient and laborious.	[72]
	Extrusion	Reduces aflatoxin contamination level by 50–80%.	Causes a loss of nutritional ingredients in the cereals.	
		Reduces the toxicity of the initial compounds.		
	Heat treatment	Kills molds attached to the surface of the cereal.	Damages cereals’ nutritional values and sensory quality.	[73]
		Destroys some natural toxins by 10–25%.	High energy consumption. Restricted by the heat resistance and moisture of cereals.	
	Ultraviolet (UV) radiation	Reduces aflatoxin concentration by up to 40–45%.	Limitations, including low penetration and narrow wavelength range, mean the industry has not yet recognized the patented process of UV inactivation of aflatoxin.	[74]
			Main degradation compound of AFB1 seems to retain residual toxicity when exposed to UV light, requiring further degrading into non-toxic forms.	
	Irradiation	High efficiency in eliminating microorganisms and other potential pathogens infecting cereals (over 68.9% for AFB1, over 51% for OTA).	High-energy consumption. Destroys water-soluble vitamins and proteins in cereals.	[75]
	Cold plasma	Does not cause heat damage to processed food or affect the protein content.	Plasma treatment of irregularly shaped or bulk food materials can be challenging.	[76]
			The potential cytotoxic effect remains unclear.	
			Amplification and continuous processing are challenges of current plasma equipment design.	
	Pulsed light	Potential substitute for traditional technology which does not reduce food quality.	Seldom able to penetrate the cereal, so it is difficult to deal with mycotoxins deep in the cereal. Reduces the germination rate of the seeds.	[75]
		Cost-effective non-thermal technology leaves no residue on food materials.		
Chemical	Fungicides and pesticides	Minimize fungal infections or insect damage to crops, reducing mycotoxin contamination by up to 55–75%.	Can produce residues hazardous to food safety and the environment.	[77]
			Fungicide concentrations tested in the laboratory exceeded the maximum solubility levels in aqueous media, so effectiveness is unclear.	
			Long-term use will produce drug resistance in molds if residue remains in the cereals, thus affecting eating quality.	
	Ozone	Can inhibit the growth, spore formation, and germination of fungi.	Antibacterial activity largely depends on the type of vegetable/fungi, growth stage, concentration, and exposure time.	[78]
		Loss of nutrition or sensory quality in food/feed is negligible.	Degradation products formed by residues are not yet fully determined.	
	Ammonia	Reducese AFs, FBs, and OTA to undetectable levels and inhibits the growth of toxin-producing molds.	Infrastructure is complex, and the European community does not allow this method to be used for human food.	[77]
	Acid treatment	Degradation of AFs (barely detectable).	Causes chemical residues, limiting its applicability in cereal due to safety concerns.	[77]

**Table 3:** Maximum tolerable level of mycotoxins in cereals and other agricultural commodities in China.

Product Name		Mycotoxin Limit (μg/kg) ^a^					
		AFB_1_	DON	OTA	ZEN	T-2	FB_1_ + FB_2_
Food category	Maize, cornmeal, maize products	≤20	≤1000	≤5.0	≤60		
	Rice, brown rice	≤10					
	Wheat, barley, other hulled cereals	≤5.0			≤60		
	Beans, bean products	≤5.0					
	Peanut, peanut products	≤20					
	Vegetable oils (except peanut and maize oil)	≤10					
	Peanut oil, maize oil	≤20					
Feed raw material	Cereals, processed products	≤30	≤500	≤100	≤1000	≤500	
	Processed maize products	≤50			≤500		≤60,000
	Vegetable oils (except peanut and maize oil)	≤10			≤1000		
	Peanut oil, maize oil	≤20					
	Other vegetative feed raw materials	≤30					
Feed products	Compound feed for piglets and young birds	≤10	≤1000		≤150		≤500
	Supplementary feed for calves and lambs	≤20			≤500		≤2000
	Other compound feed	≤20	≤3000	≤100		≤500	

**^a^**: information from GB2761-2017 and GB13078-2017.

**Table 4:** Sources of active microbial substances that detoxify mycotoxins and their conditions of detoxification.

Microbial Source	Detoxification Active Substances	Target Mycotoxin	Mycotoxin Detoxification (%)	Detoxification Conditions	Detoxification Products	Reference
*Bacillus shackletonii* L7	Bacillus aflatoxin-degrading enzyme (BADE)	Aflatoxin B_1_	92.1	37 °C	-	[129]
		Aflatoxin B_2_	84.1			
		Aflatoxin M_1_	90.4			
*Bacillus subtilis* UTBSP1	Extracellular enzymes	Aflatoxin B_1_	85.66	35–40 °C	-	[130]
*Bacillus aryabhattai* DT	Extracellular enzymes	Aflatoxin B_1_	78	37 °C	-	[131]
*Bacillus licheniformis* BL010	Two degrading enzymes (Chia010 and Lac010)	Aflatoxin B_1_	89.1	30 °C	Molecular formula is C_12_H_14_O_4_	[132]
*Bacillus velezensis* DY3108	Extracellular proteins or enzymes	Aflatoxin B_1_	91.5	80 °C, pH = 8	-	[133]
*Bacillus pimilus* E-1-1-1	Extracellular extracts	Aflatoxin M_1_	100	37 °C	-	[134]
		Aflatoxin B_1_	89.55			
*Candida versatilis* CGMCC3790	Intracellular components	Aflatoxin B_1_	70	25 °C, pH = 5.0	Molecular formulas are C_14_H_10_O_4_, C_14_H_12_O_3_, C_13_H_12_O_2_, C_11_H_10_O_4_	[116]
*Escherichia coli* CG1061	Heat-resistant protein	Aflatoxin B_1_	93.7	55 °C, pH = 8.5	Molecular formula is C_16_H_14_O_5_	[120]
*Cladosporium uredinicola* CCTCC M 2015181	Extracellular enzymes	Aflatoxin B_1_	84.50 ± 5.70	37 °C	-	[135]
*Pseudomonas aeruginosa* N17-1	Extracellular enzymes	Aflatoxin B_1_	82.8	37 °C	-	[136]
		Aflatoxin B_2_	46.8			
		Aflatoxin M_1_	31.9			
*Pseudomonas putida* MTCC 2445	Intracellular enzymes	Aflatoxin B_1_	80	50 °C, pH = 7.0	-	[137]
*Bacillus licheniformis*	CotA laccase	Aflatoxin B_1_	96	37 °C, pH = 8.0	Aflatoxin Q_1_ and *epi*-aflatoxin Q_1_	[114]
*Mycobacterium smegmatis*	MSMEG 5998 (aflatoxin-degrading F420H2-dependent reductase)	Aflatoxin B_1_	31	22 °C	-	[138]
*Pseudomonas putida* 12-3	Intracellular enzymes	Aflatoxin B_1_	83.3	30 °C, pH = 8.0	-	[139]
*Escherichia coli* 12-5		Aflatoxin B_1_	63.8			
*Tetragenococcus halophilus* CGMCC 3792	Intracellular enzymes	Aflatoxin B_1_	82.8	55 °C	Molecular formula is C_14_H_20_O_2_	[115]
*Fusarium* sp. WCQ3361	Protein	Aflatoxin B_1_	95.38	30 °C	-	[133]
*Lacticaseibacillus acidophilus* PTCC 1643	-	Aflatoxin M_1_	100	21 °C	-	[140]
*Lactiplantibacillus plantarum* PTCC 1058				37 °C		
*Flavobacterium aurantiacum*	Crude protein extracts	Aflatoxin B_1_	74.5	30 °C, pH = 7.0	-	[141]
*Phanerochaete sordida* YK-624	Manganese peroxidase (MnP)	Aflatoxin B_1_	86	30 °C, pH = 4.5	AFB_1_-8,9-dihydrodiol	[119]
*Rhodococcus erythropolis* DSM 14303	Cell-free extract	Aflatoxin B_1_	95	30 °C, pH = 7.0	-	[127]
*Nocardia corynebacterioides* DSM 12676			70			
*Nocardia corynebacterioides* DSM 20151			100			
*Mycobacterium fluoranthenivorans* sp. nov. DSM 14304			100			
*Stenotrophomonas maltophilia* 35-3	Extracellular enzymes	Aflatoxin B_1_	82.5	37 °C, pH = 8	-	[142]
*Bacillus licheniformis* CK1	Extracellular xylanase, CMCase and protease	Zearalenone	97	20 °C	-	[143]
*Clonostachys rosa* IFO 7063	Lactonohydrolase ZHD101	Zearalenone	-	pH = 7–10	1-(3,5-dihydroxy-phenyl)-10-hydroxy-1-undecen-6-one	[123]
*Bacillus subtilis* 168	Culture extract	Zearalenone	81	30 °C, pH = 8.0	-	[128]
*Bacillus natto* CICC 24640		Zearalenone	100			
*Aspergillus niger* FS10	Protease	Zearalenone	89.92	37 °C	-	[144]
*Pseudomonas putida* ZEA-1	Cell-free extract	Zearalenone	100	30–37 °C, pH = 7.0–8.0	-	[126]
*Bacillus pumilus* ES-21	Esterase	Zearalenone	95.7	40.1 °C, pH = 7.60	1-(3,5-dihydroxyphenyl)-6′-hydroxy-l′-undecen-l0′-one	[118]
*Aspergillus niger*	OTA hydrolytic enzyme	Ochratoxin A	99	37 °C, pH = 7.5	-	[145]
*Yarrowia lipolytica* Y-2	Carboxypeptidases	Ochratoxin A	97.2	28 °C	Otα	[146]
*Acinetobacter* sp. *neg1*, ITEM 17016	Carboxypeptidases	Ochratoxin A	70	37 °C	Otα	[147]
*Bacillus amyloliquefaciens* ASAG1	Carboxypeptidase	Ochratoxin A	100	37 °C, pH = 7.0	-	[148]
*Aspergillus niger*	Crude lipase (Amano A)	Ochratoxin A	100	30 °C, pH = 7.5	Otα and phenylalanine	[117]
*Aspergillus niger* W-35	Ochratoxinase	Ochratoxin A	85.1	37 °C	-	[149]
*Alcaligenes faecalis*	N-acyl-L-amina acid amidohydrolase (AfOTase)	Ochratoxin A	-	50 °C, pH = 6.5	-	[150]
*Sphingopyxis* sp. MTA144	Recombinant carboxylesterase	Fumonisin B_1_	100	30 °C, pH = 8.0	-	[151]
Bacterial consortium SAAS79	Intracellular enzymes	Fumonisin B_1_	90	28 °C, pH = 7.0	pHFB_1_a or pHFB_1_b	[121]
Bacterium ATCC 55552	Aminotransferase	Fumonisin B_1_	100	25 °C	-	[152]

## Supplementary Materials

The following supporting information can be downloaded at: https://www.mdpi.com/article/10.3390/metabo13040551/s1, Table S1: Sources of active microbial substances inhibiting toxin-producing molds and mycotoxins and their conditions of action [102,103,104,105,107,108,109,110,111,112]. References [173,174,175,176,177,178,179,180,181,182,183,184,185,186,187,188,189,190,191,192,193,194,195,196,197] are cited in the supplementary materials.
