# Supplementary material for: Mycotoxin Contamination Status of Cereals in China and Potential Microbial Decontamination Methods
Source: Metabolites. 2023 Apr 12;13(4):551. doi: 10.3390/metabo13040551 (PMC10143121; doi:10.3390/metabo13040551)
Supplement: Supplementary file 1 [file metabolites-13-00551-s001.zip › metabolites-2240832-supplementary.pdf]

SUPPLEMENTARY MATERIALS

Mycotoxin Contamination Status of Cereals in China and Potential Microbial Decontamination Methods

Jing Zhang [1,2], Xi Tang [1], Yifan Cai [1] and Wen-Wen Zhou [1,*]

[1] College of Biosystems Engineering and Food Science, Ningbo Research Institute, Zhejiang University, Hangzhou 310058, Zhejiang, China

[2] School of Chemical and Biomolecular Engineering, The University of Sydney, Sydney, NSW 2006, Australia

* Correspondence: vivianzhou11@zju.edu.cn

Table S1. Sources of active microbial substances inhibiting toxin-producing molds and mycotoxins and their conditions of action

**Microbial source**	**Active substances**	**Target mold**	**Mold inhibition (%)**	**Target mycotoxin**	**Mycotoxin inhibition (%)**	**Suppressing conditions**	**Reference**
Bacillus amyloliquefaciens UTB2	Protease	*Aspergillus parasiticus*	90	Aflatoxins	100	37°C, pH > 8	[1]
Bacillus subtilis UTB3			92	Aflatoxins	100		
Bacillus megaterium CGMCC7086	Three peptides (D1O, D1N, D2N)	-	-	Aflatoxins	70.0–80.0	28°C	[2]
Lactobacillus gasseri 1A-TV	Acidocin A and helveticin J	-	-	Aflatoxins	100	37°C	[3]
Lactiplantibacillus plantarum K35	Lactic acid, 2-butyl-4-hexyloctahydro-1H-indene, oleic acid, palmitic acid, and other substances	*Aspergillus flavus* TISTR3041	100	Aflatoxins	100	37°C, pH =6.5 ± 0.1	[4]
		Aspergillus parasiticus TISTR 3276	100	Aflatoxins	100		
Lactiplantibacillus plantarum UM55	Organic acids (lactic acid, phenyllactic acid, hydroxypenyllactic acid, and indole lactic acid)	*Aspergillus flavus* MUM 17.14	32	Aflatoxins	91	25°C, pH = 7	[5]
Lactiplantibacillus plantarum IS10	Peptides	*Aspergillus flavus* MD3	44	-	-	30°C	[6]
Lactiplantibacillus plantarum 21B	Phenyllactic and *p*-hydroxyphenyllactic acids	*Aspergillus flavus* FTDC3226	86.5 ± 5.5	-	-	26 or 30°C, pH = 4.8	[7]

Lactiplantibacillus plantarum AF1	$C_{12}H_{22}N_2O_2$	*Aspergillus flavus* ATCC 22546	-	-	-	30°C	[8]
Saccharomyces cerevisiae 117	4-Hydroxyphenethyl alcohol	*Aspergillus flavus* Z103	83	Aflatoxins	99.8	28°C	[9]
Pichia anomala WRL-076	2-phenylethanol	*Aspergillus flavus*	-	-	-	28°C	[10]
Spirulina platensis	Phenolic compounds	*Aspergillus flavus*	56	-	-	24°C	[11]
Pichia anomala ATCC 34080	Exo-chitinase, *β*-1,3-glucanase	*Aspergillus flavus*	-	-	-	28°C	[12]
Streptomyces sp.MRI142	Aflastatin A	*Aspergillus parasiticus* NRRL 2999	100	Aflatoxins	100	26°C	[13]
Streptomyces sp. SA-2581	Dioctatin A	*Aspergillus parasiticus* ATCC 26691	98	-	-	28°C	[14]
Streptomyces alboflavus TD-1	Dimethyl trisulfide and benzenamine	*Aspergillus flavus*	100	Aflatoxins	100	28°C	[15]
Streptomyces yansingensis 3-10	Reveromycins A and B	*Aspergillus flavus*	91.91	Aflatoxins	93.38	28°C	[16]

Enterbacter asburiae VT-7	1-Pentanol and Phenylethyl alcohol	*Aspergillus flavus*	100	Aflatoxins	100	28°C	[17]
Trichoderma harzianum GIM 3.442	Proteases	*Aspergillus flavus*	26.1	-	-	25°C	[18]
Pseudomonas fluorescens PB27	Chitinase	*Aspergillus flavus*	20	-	-	25°C	[19]
Candida nivariensis DMKU-CE18	1-Pentanol	*Aspergillus flavus* A39	64.90 ± 7.00	Aflatoxins	74.80 ± 6.50	28°C	[20]
Bacillus pumilus	-	*Aspergillus parasiticus* NRRL 2999	56.4	Aflatoxins	99.9	25°C, pH = 6.5	[21]
Lactobacillus sanfrancisco CB1	Organic acids	*Fusarium graminearum* 623, *Penicillium*	100	-	-	30°C	[22]
Bacillus subtilis	Peptidolipid	*Aspergillus ochraceus* SRRC 335	-	-	-	25°C	[23]
Streptomyces natalensis	Natamycin	*Aspergillus carbonarius*	-	Ochratoxin A	100	20°C	[24]
Four yeast strains (*Cyberlindnera jadinii* 273, *Candida friedrichii* 778, *Candida intermedia* 235, and	2-Phenylethanol	*Aspergillus carbonarius* and *Aspergillus ochraeus*	30.0–50.0	Ochratoxin A	56.0–74.0	25°C	[25]

Pichia anomala, P. kluyveri and *Hanseniaspora uvarum*	2-phenyl ethyl acetate	*Aspergillus ochraceus*	100	Ochratoxin A	100	30°C	[26]
Bacillus licheniformis BL350-2	3-Methy-1-butanol	*Aspergillus westerdijkiae* BA1	62	-	-	26°C	[27]
		Aspergillus carbonarius MG7	60				
		Penicillium verrucosum MC12	53				
		Aspergillus Niger MC05	50				
		Aspergillus ochraceus MD1	44				
		Aspergillus ochraceus CM5	-	Ochratoxin A	100		
Bacillus pumilus	Cyclic polypeptide or non-peptidic compound	*Aspergillus ochraceus* NRRL 3174	76	Ochratoxin A	71	25°C	[28]
Nannochloropsis sp.	Phenolic acids	*Fusarium graminearum*	67	Deoxynivalenol	100	25°C	[29]
Spirulina sp.	Phenolic acids	*Fusarium graminearum*	62	Deoxynivalenol	62	25°C	
Bacillus vallismortis ZZ185	Bacillomycin D (n-C14) and Bacillomycin D (iso-C15)	*Fusarium graminearum*	50	-	-	30°C	[30]
Ascophyum nodosum	Probiotic and seaweed extract	*Fusarium graminearum*	100	Zearalenone	100	25 ± 2°C	[31]
Pediococcus pentosaceus	Purified bacteriocin	*Fusarium graminearum*	100	Zearalenone	97.43	25 ± 2°C	[32]

Bacillus amyloliquefaciens DA12	Iturin A and volatile heptanones	*Fusarium graminearum* Z-3639	70.50 ± 1.10	-	-	30°C	[33]
		Fusarium graminearum H-11	71.30 ± 1.10				
		Fusarium graminearum H7-4	74.00 ± 2.70				
		Fusarium graminearum H7-11	72.50 ± 0.60				
Bacillus amyloliquefaciens S76-3	Iturin A and plipastatin A	*Fusarium graminearum*	100	-	-	28°C	[34]
Pediococcus acidilactici KTU05-7	Fermented permeate	-	-	Deoxynivalenol	23	32°C	[35]
Latilactobacillus sakei				Zearalenone	73	30°C	
Pediococcus pentosaceus				HT-2	58	35°C	
Pediococcus acidilactici				T-2	34	32°C	

References

1. Siahmoshteh, F.; Hamidi-Esfahani, Z.; Spadaro, D.; Shams-Ghahfarokhi, M.; Razzaghi-Abyaneh, M. Unraveling the mode of antifungal action of *Bacillus subtilis* and *Bacillus amyloliquefaciens* as potential biocontrol agents against aflatoxigenic *Aspergillus parasiticus*. *Food Control* **2018**, *89*, 300–307.
2. Chen, Y.; Kong, Q.; Liang, Y. Three newly identified peptides from *Bacillus megaterium* strongly inhibit the growth and aflatoxin B_1 production of *Aspergillus flavus*. *Food Control* **2019**, *95*, 41–49.
3. Scillato, M.; Spitale, A.; Mongelli, G.; Privitera, G.F.; Mangano, K.; Cianci, A.; Stefani, S.; Santagati, M. Antimicrobial properties of *Lactobacillus* cell-free supernatants against multidrug-resistant urogenital pathogens. *MicrobiologyOpen* **2021**, *10*, e1173.
4. Sangmanee, P.; Hongpattarakere, T. Inhibitory of multiple antifungal components produced by *Lactobacillus plantarum* K35 on growth, aflatoxin production and ultrastructure alterations of *Aspergillus flavus* and *Aspergillus parasiticus*. *Food Control* **2014**, *40*, 224–233.
5. Guimarães, A.; Santiago, A.; Teixeira, J.A.; Venâncio, A.; Abrunhosa, L. Anti-aflatoxigenic effect of organic acids produced by *Lactobacillus plantarum*. *Int. J. Food Microbiol.* **2018**, *264*, 31–38.
6. Muhialdin, B.J.; Hassan, Z.; Bakar, F.A.; Saari, N. Identification of antifungal peptides produced by *Lactobacillus plantarum* IS10 grown in the MRS broth. *Food Control* **2016**, *59*, 27–30.
7. Lavermicocca, P.; Valerio, F.; Evidente, A.; Lazzaroni, S.; Corsetti, A.; Gobbetti, M. Purification and Characterization of Novel Antifungal Compounds from the Sourdough *Lactobacillus plantarum* Strain 21B. *Appl. Environ. Microbiol.* **2000**, *66*, 4084–4090.
8. Yang, E.J.; Chang, H.C. Purification of a new antifungal compound produced by *Lactobacillus plantarum* AF_1 isolated from kimchi. *Int. J. Food Microbiol.* **2010**, *139*, 56–63.
9. Abdel-Kareem, M.M.; Rasmey, A.M.; Zohri, A.A. The action mechanism and biocontrol potentiality of novel isolates of *Saccharomyces cerevisiae* against the aflatoxigenic *Aspergillus flavus*. *Lett. Appl. Microbiol.* **2019**, *68*, 104–111.
10. Hua, S.S.T.; Beck, J.J.; Sarreal, S.B.L.; Gee, W. The major volatile compound 2-phenylethanol from the biocontrol yeast, Pichia anomala, inhibits growth and expression of aflatoxin biosynthetic genes of *Aspergillus flavus*. *Mycotoxin Res.* **2014**, *30*, 71–78.
11. Souza, M.M.d.; Prietto, L.; Ribeiro, A.C.; Souza, T.D.d.; Badiale-Furlong, E. Assessment of the antifungal activity of *Spirulina platensis* phenolic extract against *Aspergillus flavus*. *Ciênc Agrotecnol.* **2011**, *35*, 1050–1058.
12. Tayel, A.A.; El-Tras, W.F.; Moussa, S.H.; El-Agamy, M.A. Antifungal action of *Pichia* anomala against aflatoxigenic *Aspergillus flavus* and its application as a feed supplement. *J. Sci. Food Agric.* **2013**, *93*, 3259–3263.
13. Ono, M.; Sakuda, S.; Suzuki, A.; Isogai, A. Aflastatin A, a novel inhibitor of aflatoxin production by aflatoxigenic fungi. *J. Antibiot.* **1997**, *50*, 111–118.
14. Yoshinari, T.; Akiyama, T.; Nakamura, K.; Kondo, T.; Takahashi, Y.; Muraoka, Y.; Nonomura, Y.; Nagasawa, H.; Sakuda, S. Dioctatin A is a strong inhibitor of aflatoxin production by *Aspergillus parasiticus*. *Microbiology* **2007**, *153*, 2774–2780.
15. Yang, M.; Lu, L.; Pang, J.; Hu, Y.; Guo, Q.; Li, Z.; Wu, S.; Liu, H.; Wang, C. Biocontrol activity of volatile organic compounds from *Streptomyces alboflavus* TD-1 against *Aspergillus flavus* growth and aflatoxin production. *J. Microbiol.* **2019**, *57*, 396–404.
16. Shakeel, Q.; Lyu, A.; Zhang, J.; Wu, M.; Li, G.; Hsiang, T.; Yang, L. Biocontrol of *Aspergillus flavus* on Peanut Kernels Using *Streptomyces yanglinensis* 3-10. *Front. Microbiol.* **2018**, *9*, 1049.
17. Gong, A.-D.; Dong, F.-Y.; Hu, M.-J.; Kong, X.-W.; Wei, F.-F.; Gong, S.-J.; Zhang, Y.-M.; Zhang, J.-B.; Wu, A.-B.; Liao, Y.-C. Antifungal activity of volatile emitted from *Enterobacter asburiae* Vt-7 against *Aspergillus flavus* and aflatoxins in peanuts during storage. *Food Control* **2019**, *106*, 106718.

18. Deng, J.J.; Huang, W.Q.; Li, Z.W.; Lu, D.L.; Zhang, Y.; Luo, X.C. Biocontrol activity of recombinant aspartic protease from *Trichoderma harzianum* against pathogenic fungi. *Enzyme Microb. Technol.* **2018**, *112*, 35–42.
19. Akocak, P.B.; Churey, J.J.; Worobo, R.W. Antagonistic effect of chitinolytic *Pseudomonas* and *Bacillus* on growth of fungal hyphae and spores of aflatoxigenic *Aspergillus flavus*. *Food Biosci.* **2015**, *10*, 48–58.
20. Jaibangyang, S.; Nasanit, R.; Limtong, S. Biological control of aflatoxin-producing *Aspergillus flavus* by volatile organic compound-producing antagonistic yeasts. *BioControl* **2020**, *65*, 377–386.
21. Munimbazi, C.; Bullerman, L.B. Inhibition of aflatoxin production of *Aspergillus parasiticus* NRRL 2999 by *Bacillus pumilus*. *Mycopathologia* **1997**, *140*, 163–169.
22. Corsetti, A.; Gobbetti, M.; Rossi, J.; Damiani, P. Antimould activity of sourdough lactic acid bacteria: identification of a mixture of organic acids produced by *Lactobacillus sanfrancisco* CB1. *Appl. Microbiol. Biotechnol.* **1998**, *50*, 253–256.
23. Klich, M.A.; Lax, A.R.; Bland, J.M. Inhibition of some mycotoxigenic fungi by iturin A, a peptidolipid produced by *Bacillus subtilis*. *Mycopathologia* **1991**, *116*, 77–80.
24. Medina, Á.; Jiménez, M.; Mateo, R.; Magan, N. Efficacy of natamycin for control of growth and ochratoxin A production by *Aspergillus carbonarius* strains under different environmental conditions. *J. Appl. Microbiol.* **2007**, *103*, 2234–2239.
25. Farbo, M.G.; Urgeghe, P.P.; Fiori, S.; Marcello, A.; Oggiano, S.; Balmas, V.; Hassan, Z.U.; Jaoua, S.; Migheli, Q. Effect of yeast volatile organic compounds on ochratoxin A-producing *Aspergillus carbonarius* and *A. ochraceus*. *Int. J. Food Microbiol.* **2018**, *284*, 1–10.
26. Masoud, W.; Poll, L.; Jakobsen, M. Influence of volatile compounds produced by yeasts predominant during processing of Coffea arabica in East Africa on growth and ochratoxin A (OTA) production by *Aspergillus ochraceus*. *Yeast* **2005**, *22*, 1133–1142.
27. Ul Hassan, Z.; Al Thani, R.; Alnaimi, H.; Migheli, Q.; Jaoua, S. Investigation and Application of *Bacillus licheniformis* Volatile Compounds for the Biological Control of Toxigenic *Aspergillus* and *Penicillium* spp. *ACS Omega* **2019**, *4*, 17186–17193.
28. Munimbazi, C.; Bullerman, L.B. Isolation and partial characterization of antifungal metabolites of *Bacillus pumilus*. *J. Appl. Microbiol.* **1998**, *84*, 959–968.
29. Scaglioni, P.T.; de Oliveira Garcia, S.; Badiale-Furlong, E. Inhibition of in *vitro* trichothecenes production by microalgae phenolic extracts. *Food Res. Int.* **2019**, *124*, 175–180.
30. Zhao, Z.; Wang, Q.; Wang, K.; Brian, K.; Liu, C.; Gu, Y. Study of the antifungal activity of *Bacillus vallismortis* ZZ185 in *vitro* and identification of its antifungal components. *Bioresour. Technol.* **2010**, *101*, 292–297.
31. Shatha, A.; Shafiq, S. Antagonistic activity of probiotic and sea weed extract against vegetative growth for some fungi and Zearalenone production. *World J. Pharm. Res.* **2015**, *4*, 1577–1585.
32. Mahdi, L.H.; Shafiq, S.A.; Ajaa, H. Effects of crude and purified bacteriocin of *Pediococcus pentosaceus* on the growth and zearalenone production by *Fusarium graminearum*. *Int. J. Curr Eng. Technol.* **2013**, *4*, 2277–4106.
33. Lee, T.; Park, D.; Kim, K.; Lim, S.M.; Yu, N.H.; Kim, S.; Kim, H.-Y.; Jung, K.S.; Jang, J.Y.; Park, J.-C.; et al. Characterization of *Bacillus amyloliquefaciens* DA12 Showing Potent Antifungal Activity against Mycotoxigenic *Fusarium* Species. *Plant Pathol. J.* **2017**, *33*, 499–507.
34. Gong, A.-D.; Li, H.-P.; Yuan, Q.-S.; Song, X.-S.; Yao, W.; He, W.-J.; Zhang, J.-B.; Liao, Y.-C. Antagonistic Mechanism of Iturin A and Plipastatin A from *Bacillus amyloliquefaciens* S76-3 from Wheat Spikes against *Fusarium graminearum*. *PLoS ONE* **2015**, *10*, e0116871.
35. Juodeikiene, G.; Bartkiene, E.; Cernauskas, D.; Cizeikiene, D.; Zadeike, D.; Lele, V.; Bartkevics, V. Antifungal activity of lactic acid bacteria and their application for *Fusarium* mycotoxin reduction in malting wheat grains. *LWT* **2018**, *89*, 307–314.
